# Insight into uniform filming of LiF‐rich interphase via synergistic adsorption for high‐performance lithium metal anode

**DOI:** 10.1002/EXP.20230114

**Published:** 2023-12-22

**Authors:** Yufang He, Li Wang, Aiping Wang, Bo Zhang, Hiep Pham, Jonghyun Park, Xiangming He

**Affiliations:** ^1^ Institute of Nuclear and New Energy Technology Tsinghua University Beijing China; ^2^ Department of Mechanical Engineering and Aerospace Engineering Missouri University of Science and Technology Rolla, MO USA

**Keywords:** LiF‐rich solid electrolyte interphase, lithium metal anode, additive‐derived species, synergistic adsorption, film growth mechanism

## Abstract

Multi‐scale simulation is an important basis for constructing digital batteries to improve battery design and application. LiF‐rich solid electrolyte interphase (SEI) is experimentally proven to be crucial for the electrochemical performance of lithium metal batteries. However, the LiF‐rich SEI is sensitive to various electrolyte formulas and the fundamental mechanism is still unclear. Herein, the structure and formation mechanism of LiF‐rich SEI in different electrolyte formulas have been reviewed. On this basis, it further discussed the possible filming mechanism of LiF‐rich SEI determined by the initial adsorption of the electrolyte‐derived species on the lithium metal anode (LMA). It proposed that individual LiF species follow the Volmer–Weber mode of film growth due to its poor wettability on LMA. Whereas, the synergistic adsorption of additive‐derived species with LiF promotes the Frank‐Vander Merwe mode of film growth, resulting in uniform LiF deposition on the LMA surface. This perspective provides new insight into the correlation between high LiF content, wettability of LiF, and highperformance of uniform LiF‐rich SEI. It disclosed the importance of additive assistant synergistic adsorption on the uniform growth of LiF‐rich SEI, contributing to the reasonable design of electrolyte formulas and high‐performance LMA, and enlightening the way for multi‐scale simulation of SEI.

## Graphical abstract

The LiF wettable and lithiophilic additive‐derived species induce synergistic adsorption with LiF, promoting tiny LiF deposition on the lithium metal anode surface, which provides new insight into the correlation between high LiF content, wettability of LiF, and high‐performance LiF‐rich solid electrolyte interphase.

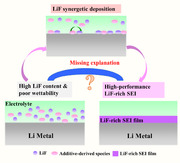

## 1 INTRODUCTION

Digital twin enables rational design of advanced batteries, as well as efficient full‐life application and safety management of batteries.^[^ ^1^, ^2^, ^3^ ^]^ Solid electrolyte interphase (SEI) on the anode surface is essential for lithium‐ion/lithium‐metal batteries (LIBs/LMBs) with good cycling stability, rate capability, temperature tolerance, and even thermal safety.^[4]^ In this sense, the simulation of the SEI filming process is an important part of the digital twin.^[^ ^5^, ^6^, ^7^ ^]^

Lithium metal anode (LMA) is regarded as the ultimate anode that the energy density of rechargeable batteries approaches its limit. With the various application situation, the requirements for the specific capacity of the batteries keep increasing. Therefore, many researchers focus on designing LMA with a higher electrochemical performance. It is well accepted that the SEI is generally a deposition layer consisting of electrolyte‐derived species during the first charge, and LiF‐rich SEI is vital for a lithium metal anode with high performance.^[^ ^6^, ^8^, ^9^, ^10^, ^11^ ^]^ Therefore, tremendous efforts have been devoted to interphase engineering^[^ ^12^, ^13^, ^14^, ^15^, ^16^ ^]^ and electrolyte formula modification^[^ ^17^, ^18^, ^19^, ^20^, ^21^, ^22^ ^]^ to achieve a high‐performance LiF‐rich SEI on the lithium‐metal anode (LMA). An artificial SEI via interphase engineering is adapted to improve the performance of SEI film,^[^ ^23^, ^24^, ^25^, ^26^, ^27^ ^]^ such as fabricating lithiophilic lithium alloy on the LMA surface to guide the uniform deposition and growth of SEI components. Whilst, designing electrolyte formulas^[^ ^28^, ^29^, ^30^, ^31^, ^32^ ^]^ can effectively regulate the electrolyte‐derived species and consequently control the film growth process and the uniformity of SEI, which is a practically accessible approach to improving the safety and cycling performance of LMBs.

The composition and structure determine the ionic transport of SEI, which is vital for the reaction kinetics of LMBs. The Li‐ion diffusion in LiF crystals is slow, which conflicts with the high ionic conductivity of LiF‐rich SEI.^[^ ^33^, ^34^ ^]^ However, it claimed that the distribution of inorganic components in SEI is with nano‐tiny grain and that there exists tremendous grain boundaries. The Li‐ion diffuses much faster in the grain boundary than in the bulk of inorganic components.^[35]^ In addition, it also demonstrated that the Li‐ion diffuses faster in the grain boundary of LiF/Li_2_O than in the grain boundary of pure LiF or Li_2_O. Therefore, the distribution of the SEI components is crucial for Li‐ion transport behavior, affecting the electrochemical performance of SEI.^[^ ^35^, ^36^ ^]^

**FIGURE 1:**
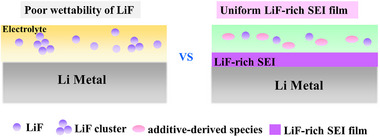
Schematic of the contradiction between the poor wettability of LiF and the high performance of the uniform LiF‐rich SEI film.

Although LiF components are believed to be beneficial for the high performance of SEI, it is neglected that the poor wettability of LiF on Li metal indicates it is difficult for LiF to form a uniform and thin film (Figure 1). It was reported that the high interfacial energy of LiF on the LMA surface leads to the minimum interface area of LiF/Li^[^ ^37^, ^38^, ^39^, ^40^ ^]^ and instability in the adsorption of individual LiF on the LMA surface.^[^ ^41^, ^42^ ^]^ Therefore, there exists a contradiction between the poor wettability of LiF and a uniformly dense LiF‐rich SEI film. Herein, the electrolyte formulas with additives facilitating the formation of LiF‐rich SEI and current cognitions of LiF‐rich SEI are systematically reviewed. Then, the possible effect of the additive‐derived species on the initial filming process is discussed. A new understanding of the formation and structure of LiF‐rich SEI seems reasonable to unveil the similarities and smallish differences behind various experimental progress. This work bridges the gap between electrolyte‐derived species and the SEI layer with spatial and compositional structure. It helps to enlighten the way towards full‐scale modeling of the SEI layer, as well as rational design and optimization of electrolyte formulas to obtain more effective LiF‐rich SEI, and consequently improve the practical application of LMA.

## 2 THE SIGNIFICANCE OF LiF‐RICH SEI ON LMA

LiF is regarded as a beneficial SEI component to realize dendrite‐free LMA owing to its high interfacial energy against LMA, low electronic conductivity,^[^ ^19^, ^43^, ^44^, ^45^, ^46^ ^]^ high mechanical strength,^[47]^ and ultralow solubility.^[^ ^48^, ^49^, ^50^ ^]^ For example, the SEI layer is rich in LiF in LiPF_6_/EMC/FEC/LiTFSI/LiDFOB electrolyte,^[48]^ which mainly derives from FEC and LiPF_6_. The Li||Li[Ni_0.59_Co_0.2_Mn_0.2_Al_0.01_]O_2_ cell with a stable LiF‐rich SEI formed on the LMA surface keeps a 75% specific discharge capacity of 177 mAh/g after 500 h and the Coulombic efficiency is greater than 99.3%. In addition, in LiPF_6_/EC/DEC/FEC electrolyte,^[51]^ the FEC additive induces the SEI layer to enrich in LiF, which increases the Coulombic efficiency up to 98% after 100 cycles in Li||Cu cell, and shows a high initial capacity of 154 mAh/g in Li||LiNi_0.5_Co_0.2_Mn_0.3_O_2_ (NMC) cell. Furthermore, in a concentrated LiTFSI/LiDFOB/DME electrolyte,^[52]^ a stable LiF‐rich SEI formed on the LMA surface realizes a capacity retention of Li||LiNi_1/3_Mn_1/3_Co_1/3_O_2_ up to 90% for 300 cycles. Moreover, the Li||NCM622 cell with LiPF_6_/EMC/FEC electrolyte‐derived LiF‐rich SEI exhibits a high initial capacity of 1.8 mAh/cm^2^ with excellent cycling stability and a high Coulombic efficiency of 99.8% for 500 cycles.^[53]^ Through reviewing literature related to the performance improvement of LMA, most works focus on the performance of electrolyte‐derived LiF‐rich SEI. However, a few papers concentrate on the structure of LiF‐rich SEI and even less work emphasizes the film formation mechanism of LiF‐rich SEI.

## 3 CURRENT COGNITION ON LiF‐RICH SEI

According to current cognition on SEI, uniform and dense SEI is necessary to ensure highly reversible lithium plating/stripping. Although different lithium salts, F‐contained solvents, and additives are applied to produce LiF and achieve a high‐performance LiF‐rich SEI film, numerous works experimentally validate that only abundant production of LiF from electrolyte decomposition cannot guarantee a high‐performance LiF‐rich SEI. Various additives are generally incorporated into the electrolyte to assist the formation of thin and uniform SEI film. For instance, the LiF‐rich SEI can be formed on the LMA surface in LiPF_6_/FEC/EMC electrolyte. After incorporating LiDFBOP, the resistance of the electrolyte‐derived LiF‐rich SEI decreases from 40 to 16 Ω^[54]^ and the average Coulombic efficiency of LMA increases from 92.0% to 96.7%. Therefore, there exist additional effects of electrolyte additives on the electrochemical performance of LiF‐rich SEI. Abundant inorganic salts/additives, including LiNO_3_,^[43,^ ^55^, ^56^, ^57^ ^]^ LiDFOB,^[^ ^58^, ^59^ ^]^ LiBOB,^[^ ^58^, ^60^ ^]^ LiDFBOP,^[^ ^54^, ^61^ ^]^ LiBODFP,^[62]^ LiPF_6_,^[18,^ ^63^ ^]^ and LiPO_2_F_2_^[64,^ ^65^ ^]^ are incorporated to enhance the electrochemical performance of LiF‐rich SEI. For instance, the interfacial resistance of the Li||LiFePO_4_ coin cell with LiPF_6_/EC/DEC electrolyte increases by 21 Ω. After incorporating FEC and LiNO_3_ additives, the resistance increases only 4 Ω after 100 cycles and the LiPF_6_/DME/FEC/LiNO_3_ electrolyte‐derived LiF‐rich SEI realizes Coulombic efficiency up to 99.96 %.^[56]^ Besides, the SEI resistance is 156 Ω in LiPF_6_/EC/DMC electrolyte, while it decreases to 35 Ω after involving FEC/LiNO_3_/sulfolane (SL) additives and enabling the average Coulombic efficiency up to 99.6% in 100 cycles.^[43]^

Above research indicates that the interaction between electrolyte‐derived species and LiF is crucial for LiF‐rich SEI with high performance. The considerable discrepancy in the electrochemical performance of similar LiF‐rich SEI components might relate to the existing status and distribution of LiF in the LiF‐rich SEI, which is associated with film formation process. As mentioned, the poor wettability and low ionic conductivity of LiF imply that individual LiF components can hardly form a uniform and high ionic conductive SEI. This is in contrast with the fact that LiF‐rich SEI feature uniform and thin morphology, as well as low resistance. The contradiction between the intrinsic property of LiF and the property of LiF‐rich SEI promotes us to focus on the film formation process of SEI film.

The electrolyte‐derived in‐situ LiF‐rich SEI film formed on the LMA interface via precipitation of the chemical or electrochemical reaction products. Through reviewing literature, the merits of LiF‐rich SEI over common SEI layer is that the LiF has high interface energy against LMA, fast ionic transportation, low electronic conductivity, and good stability, which ensures high specific capacity and high Coulombic efficiency of LMBs. The components and their distribution in LiF‐rich SEI and the film formation process in various electrolytes lead to the electrochemical discrepancy. Therefore, in this section, the characterization, and the formation mechanisms of LiF‐rich SEI in different electrolyte formulas are reviewed.

### 3.1 The structure of LiF‐rich SEI

The structure of LiF‐rich SEI and its physicochemical properties have been investigated via various analytical techniques, which include ex situ and in situ.^[^ ^66^, ^67^, ^68^, ^69^, ^70^, ^71^, ^72^, ^73^, ^74^, ^75^, ^76^, ^77^ ^]^ As shown in Table 1, the X‐ray photoelectron spectroscopy (XPS),^[^ ^42^, ^71^, ^72^, ^75^, ^78^, ^79^, ^80^, ^81^ ^]^ electron energy loss spectroscopy (EELS),^[74]^ energy dispersive X‐ray spectroscopy (EDX),^[71]^ and time of flight secondary ion mass spectrometry (ToF‐SIMs)^[^ ^43^, ^56^, ^81^ ^]^ are applied to analyze SEI chemical species, elemental ratios, component distribution, and preferential anion decomposition. In addition, scanning electron microscopy (SEM)/transmission electron microscopy (TEM)/high‐resolution transmission microscopy (HRTEM),^[^ ^58^, ^80^ ^]^ cryogenic TEM/HRTEM^[^ ^42^, ^73^, ^74^, ^75^, ^80^, ^82^, ^83^, ^84^ ^]^ can probe the SEI morphology, the distribution of inorganic components and the amorphous polymeric matrix. Furthermore, cryo‐STEM EELS/EDX^[^ ^42^, ^74^, ^75^, ^84^ ^]^ identify the chemical composition, element distribution, and bonding environment. Lastly, atomic force microscopy (AFM),^[^ ^74^, ^80^, ^81^ ^]^ electrochemical quartz crystal microbalance (EQCM),^[^ ^77^, ^78^ ^]^ differential electrochemical mass spectrometry (DEMS),^[^ ^75^, ^77^ ^]^ and electrochemical impedance spectrum (EIS)^[73]^ are employed to investigate the growth mode of SEI, electrochemical stability, and ionic transport properties of SEI.

**TABLE 1:** Analytical techniques used for characterization of SEI.

Analytical techniques	SEI layer information
(XPS),^[^ ^42^, ^71^, ^72^, ^75^, ^78^, ^79^, ^80^, ^81^ ^]^ EELS,^[74]^ EDX,^[71]^ and ToF‐SIMs^[^ ^43^, ^56^, ^81^ ^]^	Chemical species, elemental ratios, components distribution, and preferential anion decomposition
SEM/TEM/HRTEM,^[^ ^58^, ^80^ ^]^ cryo‐TEM/HRTEM^[^ ^42^, ^73^, ^74^, ^75^, ^80^, ^82^, ^83^, ^84^ ^]^	Ordered and non‐ordered composition distribution, distribution of nanocrystalline inorganic components and the amorphous polymeric matrix
cryo‐STEM EELS/EDX^[^ ^42^, ^74^, ^75^, ^84^ ^]^	Chemical species, element distribution, and bonding environment
AFM^[^ ^74^, ^80^, ^81^ ^]^, EQCM,^[^ ^77^, ^78^ ^]^ DEMS^[^ ^75^, ^77^ ^]^ and EIS^[73]^	Growth mode of SEI, quantify SEI dissolution, electrochemical stability, ionic transport property

Abbreviations: AFM, atomic force microscopy; cryo‐TEM, cryogenic; DEMS, differential electrochemical mass spectrometry; EDX, energy dispersive X‐ray spectroscopy; EELS, electron energy loss spectroscopy; EIS, electrochemical impedance spectrum; EQCM, electrochemical quartz crystal microbalance; HRTEM, high‐resolution transmission microscopy; SEM, scanning electron microscopy; ToF‐SIMs, time of flight secondary ion mass spectrometry; XPS, X‐ray photoelectron spectroscopy.

The cryo EDX and F 1s spectra of XPS show that the SEI is rich in F, S, and O in the 1 m LiFSI/FDMB,^[71]^ indicating it is an anion‐derived SEI. It proposes that the Li‐F interaction and increased FSI^−^/solvent ratio in the Li‐ion solvation sheath significantly improve the stability of the Li metal anode. However, the LiF existence status and distribution are not discussed. The cryo‐TEM demonstrates that the SEI includes amorphous organic species and crystalline LiF in the 1 m LiPF_6_ in EC/EMC.^[82]^ In addition, the XPS depth profiling on 1.0 m LiPF_6_/EC/DEC with FEC additive shows that the outer layer of SEI is rich in carbon‐oxygen bonding species.^[42]^ With increasing depth, the Li_2_O and LiF increase and rich in the inner layer of SEI. However, the cryo high‐resolution TEM and cryo‐STEM EELS mapping of the Li, O, and F K‐edges displays that the LiF is absent in the compact layer of SEI even in a highly fluorinated FEC‐containing electrolyte. Because the solubility of LiF is higher than Li_2_O carbonate electrolytes and it prefers to precipitate on the active material surface as nanoparticles.^[42]^ Furthermore, the cryo‐TEM combined with in‐depth XPS, EDS, AFM, and SEM demonstrates that the SEI is multilayer and the LiF is rarely observed in the compact SEI layer and it is agglomerated as nanoparticles.^[^ ^58^, ^80^, ^85^ ^]^

Cui et al. attempt to identify SEI components and their existence status with synchrotron‐based X‐ray diffraction and pair distribution function analysis.^[86]^ The SEI includes rich LiH and LiF in LiFSI/PC/DMC/DME electrolytes. It first claimed that LiH may be misidentified as LiF in previous works. Compared to LiF in the bulk phase, LiF in the SEI has a larger lattice parameter and smaller grain size, contributing to high Li‐ion transport. It explained that LiF, as an ionic insulator, is a beneficial component for the SEI. In addition, Cui et al. also propose that some factors may cause error and misinterpretation of SEI when using XPS, including poorly rinsed SEI samples, the chemical signature of the SEI varying in the *x–y* plane, and chemical changes of SEI under the ultrahigh vacuum of XPS instruments or Ar ion sputtering.^[79]^ Furthermore, the SEI dissolution in the electrolyte will affect the existing status and distribution of LiF, which determines SE film formation and growth.^[87]^ The EQCM, XPS, and nuclear magnetic resonance (NMR) spectroscopy revealed that the LiF‐rich SEI solubility depends on not only its composition but also the solvent in electrolyte.^[^ ^77^, ^78^, ^87^ ^]^ Therefore, based on the literature review, the existing status and distribution of LiF in different electrolyte formulas characterized by different advanced technologies are still controversial. Whether LiF is in the compact layer of the SEI, LiH in the SEI being misidentified as LiF, or LiF being dissolved in the electrolyte and distributed as nanoparticles on the LMA surface still requires more convincing evidence for clarification.

### 3.2 The formation mechanism of LiF‐rich SEI

According to previous works, SEI components and their distribution vary with electrolyte formulas. Additives, with a small ratio in the electrolyte, lead to significant improvement in the performance of LiF‐rich SEI. There are two reasons behind this: one is the additives or its derived species promotes the production of LiF. The other is that the additives or their derived species change the film growth mechanism of LiF‐rich SEI. Currently, the reaction mechanism has been studied extensively. However, the film growth mechanism of LiF‐rich SEI is neglected. There is no understanding of the confliction that LiF is unwettable toward LMA while it forms a thin but dense LiF‐rich SEI layer on LMA. For example, in the 1 M LiPF_6_/EC/DMC electrolyte, LiF‐rich SEI becomes more uniform after incorporating sulfolane, FEC, and LiNO_3_ additive as the reduction of FEC and LiNO_3_ in the solvation sheath of Li‐ion produces abundant additive‐derived LiF, Li_3_N and LiN_x_O_y_ species.^[^ ^43^, ^56^, ^88^, ^89^ ^]^ It speculated that the simultaneous decomposition of FEC, NO_3_^−^ (or PF_6_^−^) facilitated the uniform dispersion of LiF, Li_3_N, and LiN_x_O_y_, which resulted in faster ionic transportation^[^ ^43^, ^56^, ^90^ ^]^ and eventually improved the fast charging and long‐life of LMBs. In addition, in the LiPF_6_/EMC/FEC/LiDFBOP electrolyte, the FEC and LiDFBOP participate in the solvation sheath of Li‐ion, which can be preferentially reduced on the LMA surface at high potential. Compared with LiPF_6_/FEC/EMC electrolyte, the synergistic effect of FEC with LiDFBOP in the LiPF_6_/FEC/EMC/LiDFBOP electrolyte, produced the derived species LiF and Li_x_PO_y_F_z_, leading to faster Li‐ion transportation and better stability of LiF‐rich SEI. Therefore, it proposed that incorporating additional additives, such as FEC, LiNO_3_, and LiDFBOP, is beneficial to form a compact and uniform LiF‐rich SEI layer via modifying the solvation sheath of Li‐ion. In contrast, a non‐uniform SEI is formed in the electrolyte without additives, which results in non‐uniform Li‐ion flux and Li deposition. Except for inorganic electrolyte‐derived species, the impact of organic electrolyte‐derived species on the film formation process is also ignored by previous works. It demonstrated that the organic electrolyte composition FEC reduces to VC and LiF, followed by subsequent VC reduction, promoting the formation of LiF‐rich SEI at high reduction potential^[^ ^19^, ^49^, ^51^, ^91^, ^92^, ^93^ ^]^ and improving SEI flexibility.^[^ ^94^, ^95^, ^96^, ^97^ ^]^ The VC further decomposes into HCO_2_Li, Li_2_C_2_O_4_, Li_2_CO_3_, and Poly(VC).^[^ ^98^, ^99^, ^100^ ^]^ Therefore, the organic decomposition products, such as poly (VC), might also contribute to the uniformity and stability of SEI.

The previously claimed mechanism about additives is participating in the solvation sheath of Li‐ion and producing abundant additive‐derived species, either LiN_x_O_y_ or Li_x_PO_y_F_z_, enabling Li‐ion highly conductive SEI by constructing a fast Li‐ion transport pathway.^[^ ^43^, ^54^, ^56^ ^]^ However, if the conductivity and wettability are locally uneven, it may eventually cause a non‐uniform electrochemical reaction and accelerate the lithium dendrite growth. Hu et al. provided an explanation regarding the function mechanism of additive‐derived species on the high Li‐ion conductivity of SEI.^[101]^ That is, the ionic conductivity of SEI can be improved significantly by designing the composite Li_2_CO_3_ and LiF at the atomic scale. The existence of Li_2_CO_3_ can suppress the formation of large‐sized bulk‐phase LiF and promote uniform distribution of LiF and Li_2_CO_3_ components in SEI, creating a beneficial local environment for Li‐ion diffusion.

**FIGURE 2:**
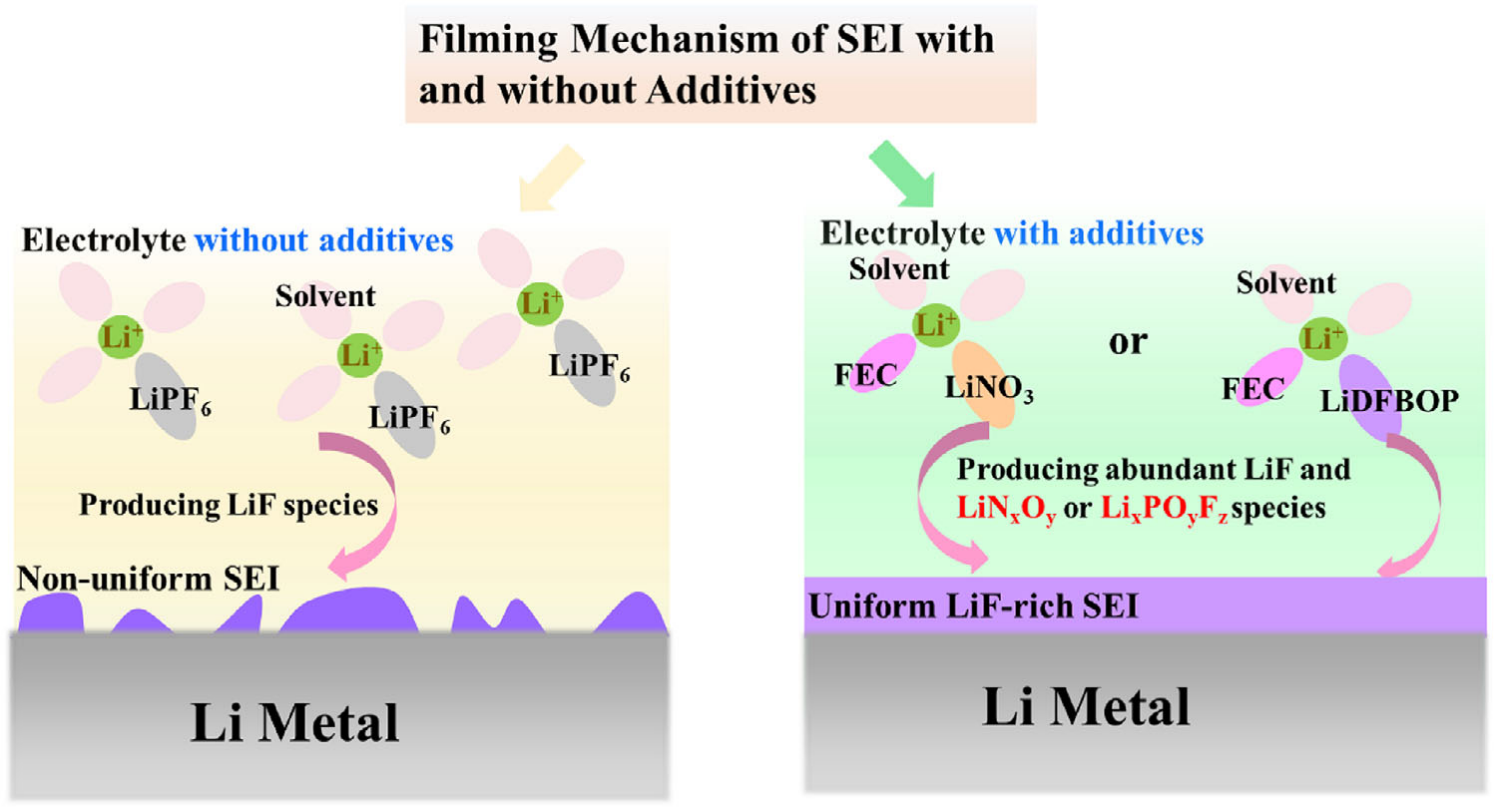
A non‐uniform SEI forms on the LMA surface owing to the poor wettability of LiF towards LMA in the electrolyte without effective film‐forming additives. Whereas, the additive‐derived species participating in the SEI film formation process in the electrolyte with effective film‐forming additives alter the existence and distribution of LiF in SEI and promote uniform filming of SEI.

Therefore, the distribution and existence state of LiF in SEI, which resulted from the film growth process of SEI with additive participation, has a more significant influence on the performance of SEI. As shown in Figure 2, a non‐uniform SEI formed on the LMA surface owing to the poor wettability of LiF towards LMA in the electrolyte without effective film‐forming additives. Whereas, the synergistic precipitation of the additive‐derived species (such as Li*_x_* NO*_y_*, and Li*_x_* PO*_y_* F*_z_*) with LiF enhances uniform LiF filming in a composite mode in the electrolyte with effective film‐forming additives, and the composite film well coincides with the structure model reported by Hu et al.^[101]^ Due to the limitations and challenges in interface characterization, the investigation of SEI film quality from a theoretical perspective is necessary. Therefore, the possibility of synergistic precipitation between LiF and additive‐derived species only at the very beginning of the filming process via first‐principles calculations will be discussed in the next section.

## 4 DISCUSSION ON POSSIBLE FILMING MECHANISM OF LiF‐RICH SEI

Although enormous efforts have been devoted to uncovering the chemical intrinsic, structure, and ionic transport of SEI, the initial nucleation and growth mode of SEI are still unclear. The initial formation process of LiF‐rich SEI is not only the premise of understanding its physicochemical property but also determining the structure and morphology of LiF‐rich SEI, which is crucial for the long cycling performance of LMBs.^[81]^ As illustrated in Figure S1, Supporting Information, there are mainly three film formation modes. The Volmer‐Weber mode (VWM) is that the insoluble component has a poor affinity for the substrate and prefers to accumulate together to form nucleation clusters. As the number of clusters increases, the clusters will precipitate as isolated nuclei and then further grow into three‐dimensional islands. The Frank–Vander Merwe mode (FVDM) is that the insoluble component has a good affinity to the substrate, and the interaction between the insoluble component and substrate is stronger than the interaction between two insoluble components. The Stranski–Krastanov mode (SKM) is the combination of the VWM and FVDM, following the FVDM first and then altering to the VWM.

**FIGURE 3:**
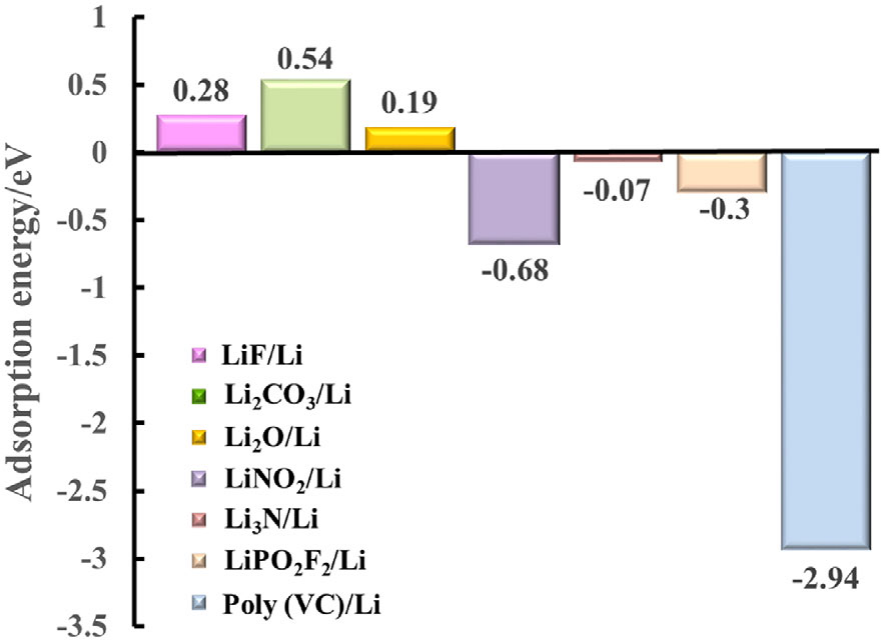
The adsorption energy of LiF, Li_2_CO_3,_ Li_2_O, LiNO_2_, Li_3_N, LiPO_2_F_2_, and Poly(VC) on the LMA surface are calculated. It indicates that individual LiF, Li_2_CO_3_, and Li_2_O cannot stably adsorb on the LMA surface, while LiNO_2_, Li_3_N, LiPO_2_F_2_, and Poly(VC) can adsorb firmly.

According to the growth mechanisms for heterogeneous film, easy adsorption of electrolyte‐derived species on the LMA surface leads to uniform growth of SEI film with a lower energy barrier for nucleation, as described in Frank–Vander Merwe mode (FVMM). However, if the electrolyte‐derived species cannot adsorb stably on the surface of LMA, it needs to overcome a high energy barrier for nucleation, as described with Volmer–Weber mode (VWM). The nuclei or clusters form in the electrolyte and then deposit on the surface of LMA as nucleation, leading to a non‐uniform SEI film. Therefore, the adsorption energy of individual LiF, Li_2_CO_3_, Li_2_O, LiNO_2_, Li_3_N, LiPO_2_F_2_, and Poly(VC), which are representative electrolyte‐derived species according to literatures,^[^ ^25^, ^44^, ^47^ ^]^ on LMA surfaces is calculated (Figure S2, Supporting Information). The adsorption energies of LiF/Li, Li_2_CO_3_/Li, Li_2_O/Li, LiNO_2_/Li, Li_3_N/Li, LiPO_2_F_2_/Li, and Poly(VC)/Li are 0.28, 0.54, 0.19, −0.68, −0.07, −0.3 and −2.94 eV, respectively (Figure 3), indicating that individual LiF, Li_2_CO_3_, and Li_2_O cannot stay stably on the LMA surface while LiNO_2_, Li_3_N, LiPO_2_F_2_, and Poly(VC) are lithiopholic.

**FIGURE 4:**
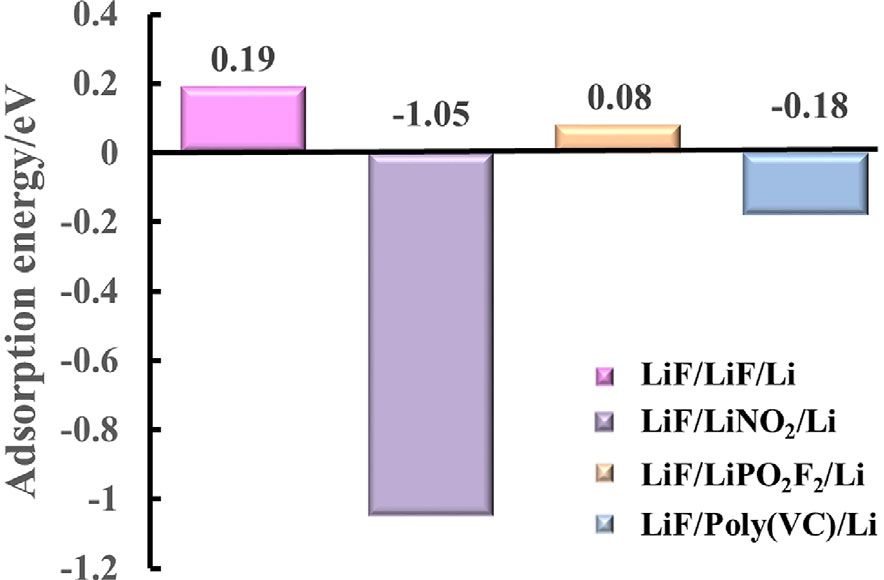
The adsorption energy of LiF/LiF/Li, LiF/LiNO_2_/Li, LiF/LiPO_2_F_2_/Li, and LiF/Poly(VC)/Li on the lithium metal surface. The synergistic deposition of electrolyte‐derived inorganic LiN*_x_* O*_y_*, Li*_x_* PO*_y_* F*_z_*, and organic poly(VC) species with LiF can be deduced. The synergistic deposition accelerates LiF precipitation as a uniform composite, leading to a uniform and dense LiF‐rich SEI.

Based on the reported results and our calculations on the adsorption energy of individual electrolyte‐derived inorganic and organic species on LMA surface, it confirms that the most common inorganic species (LiF, Li_2_CO_3_, and Li_2_O) in SEI can hardly form uniform layer, and the lithiopholic electrolyte‐derived species (LiN*_x_* O*_y_*_,_ Li*_x_* PO*_y_* F_z_, and Poly(VC)) may be adsorbed before LiF, Li_2_CO_3_, and Li_2_O. If the lithiopholic species are adsorbed on the LMA surface and creating favorable adsorption sites for lithiphobic species, it is understandable that the lithiophobic species tend to precipitate beside the lithiopholic species, resulting in synergetic deposition. Accordingly, the structures of LiF/LiF/Li, LiF/LiNO_2_/Li, LiF/LiPO_2_F_2_/Li, and LiF/Poly(VC)/ Li are constructed (Figure S3a, Supporting Information) to calculate the adsorption of LiF on LMA surface. The adsorption energy of LiF on LiF/Li, LiNO_2_/Li, LiPO_2_F_2_/Li and Poly(VC)/Li surface are 0.19, −1.05, 0.08, and −0.18 eV, respectively (Figure 4), implying that the LiNO_2_, LiPO_2_F_2_, and Poly(VC) species facilitate LiF adsorption/precipitation. In detail, the lithiophilic LiNO_2_, LiPO_2_F_2_, and Poly(VC) species provide favorable sites for LiF deposition on the LMA surface via the Li‐O bond (Figure S3b, Supporting Information). Easy LiF adsorption on LMA due to the lithiophilic species facilitates compact and uniform LiF‐rich SEI formation. Then it can be concluded that it is the synergistic deposition of electrolyte‐derived lithiophilic LiN*_x_* O*_y_*, Li*_x_* PO*_y_* F*_z_*, and Poly(VC) species with LiF that enables uniform LiF‐rich composite deposition on the LMA surface during the initial film formation process, and consequently the formation of a compact and uniform LiF‐rich SEI.

**FIGURE 5:**
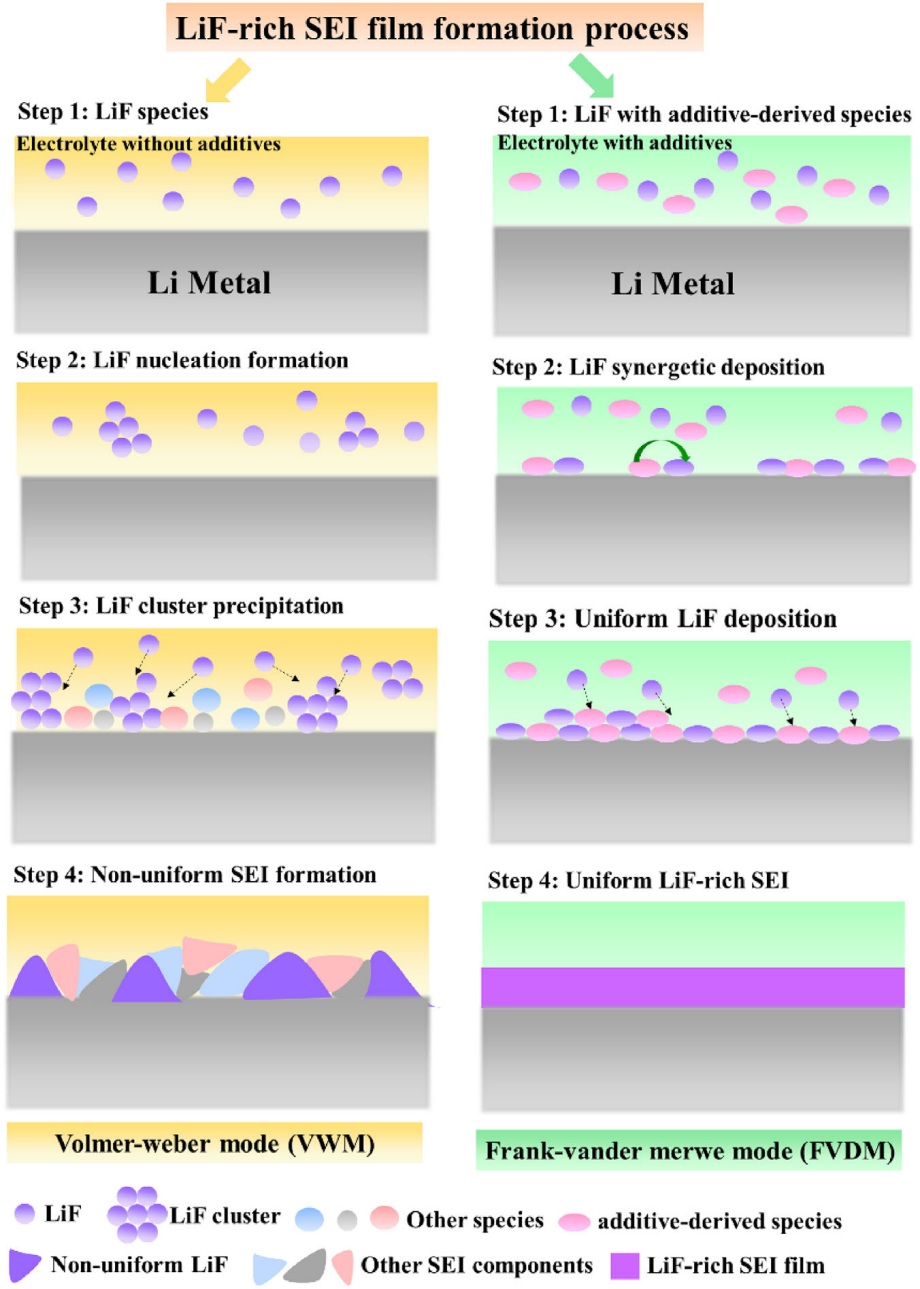
Schematic formation process LiF‐rich SEI film with and without synergetic deposition. LiF without synergetic deposition prefers to deposit as isolated LiF nucleation, and the film growth tends to follow VWM. Whereas, the synergistic deposition due to lithiopholic and LiF‐wettable electrolyte‐derived species leads to LiF‐rich composite deposition and composite film growth in FVDM.

The initial adsorption process greatly influences the following film growth mode of SEI, which is directly related to the structure, morphology, and uniformity of LiF‐rich SEI. Individual LiF on the LMA surface may follow the VWD of film growth due to its lithiophobic property. As shown in Figure 5, individual LiF is difficult to adsorbed stably on the LMA surface, it prefers to aggregate together to form nuclei or clusters in the electrolyte and finally precipitate on the LMA surface when the total surface energy of nuclei/clusters is high enough. On the contrary, the synergistic deposition of LiF with lithiophilic electrolyte‐derived species (such as LiN_x_O_y_, Li_x_PO_y_F_z_, and Poly(VC)) makes LiF‐rich film grow in the FVDM mode, resulting in LiF highly dispersed LiF‐rich composite film. Considering LiN_x_O_y_, Li_x_PO_y_F_z_ and Poly(VC) present different binding capabilities towards LMA and LiF, careful and deliberate design of the electrolyte formulas is needed to tune the film growth mode and finally the morphology, components, structure and performance of LiF‐rich SEI.

## 5 SUMMARY

LiF‐rich SEI is necessary for LMA with excellent cycling stability and rate capability. It is generally believed that the high LiF content in SEI contributes to high‐performance SEI film. However, the poor wettability of LiF on lithium metal is contradictory to the uniform and dense properties of LiF‐rich SEI, indicating a lack of understanding and modeling of the LiF‐rich SEI. Based on a comprehensive review of current cognition on the LiF‐rich SEI, including the composition, structure, film formation mechanism and optimized electrolyte formulas, the possibility of ‘synergistic precipitation’ mechanism is discussed. This ‘synergistic precipitation’ mechanism seems to be not only unlocking the conflicts (poor wettability of LiF on LMA vs dense and uniform LiF‐rich SEI, and low Li‐ion conductivity of LiF versus high Li‐ion conductivity of LiF‐rich SEI), but also well addressing the function of electrolyte additives. In detail, LiF without synergetic adsorption tends to follow VWM of film growth. The content and dispersity of LiF in SEI are low. On the contrary, lithiopholic and LiF wettable species (such as the electrolyte‐derived LiN_x_O_y_, Li_x_PO_y_F_z_, and Poly(VC)) on LMA lead to synergistic precipitation with LiF, promoting LiF deposition as a lithiopholic composite. This facilitates SEI growth following FVDM of film growth and results in compact and uniform film. This perspective offers new insight into SEI film construction. Synergistic adsorption effect can alter the adsorption and deposition behavior of target species (such as LiF) on the LMA surface, which provides a new strategy for electrolyte design and facilitates the development of safe, fast‐charging, and long‐life LMBs.

## AUTHOR CONTRIBUTIONS

Yufang He wrote the draft of the perspective and performed theoretical simulations. Xiangming He and Li Wang provided guidance and ideas for this work. Jonghyun Park and Hiep Pham modified the manuscript. Aiping Wang and Bo Zhang provided suggestions for the calculations and manuscript.

## CONFLICT OF INTEREST STATEMENT

The authors declare no conflicts of interest.

## Supporting information

Supporting Information

## References

[1] H. Zhang , D. Ren , H. Ming , W. Zhang , G. Cao , J. Liu , L. Wang , J. Song , J. Qiu , J. Wang , X. He , H. Zhang , Adv. Energy Mater. 2023, 13, 2202660.

[2] Y. Wang , J. Tian , Z. Sun , L. Wang , R. Xu , M. Li , Z. Chen , Renewable Sustainable Energy Rev. 2020, 131, 110015.

[3] S. Tamilselvi , S. Gunasundari , N. Karuppiah , A. Razak RK , S. Madhusudan , V. M. Nagarajan , T. Sathish , M. Z. M. Shamim , C. A. Saleel , A. Afzal , Sustainability 2021, 13, 10042

[4] W. Cao , Q. Li , X. Yu , H. Li , eScience 2022, 2, 47.

[5] D. Diddens , W. A. Appiah , Y. Mabrouk , A. Heuer , T. Vegge , A. Bhowmik , Adv. Mater. Interfaces 2022, 9, 2101734.

[6] A. Wang , S. Kadam , H. Li , S. Shi , Y. Qi , npj Comput. Mater. 2018, 4, 15.

[7] P. Yu , Q. Sun , Y. Liu , B. Ma , H. Yang , M. Xie , T. Cheng , ACS Appl. Mater. Interfaces 2022, 14, 7972. 35129322 10.1021/acsami.1c22610

[8] X.‐B. Cheng , R. Zhang , C.‐Z. Zhao , F. Wei , J.‐G. Zhang , Q. Zhang , Adv. Sci. 2016, 3, 1500213. 10.1002/advs.201500213 PMC5063117 27774393

[9] R. Wang , W. Cui , F. Chu , F. Wu , J. Energy Chem. 2020, 48, 145.

[10] H. G. Lee , S. Y. Kim , J. S. Lee , npj Comput. Mater. 2022, 8, 103.

[11] H. Adenusi , G. A. Chass , S. Passerini , K. V. Tian , G. Chen , Adv. Energy Mater. 2023, 13, 2203307.

[12] C. Liu , Z. G. Neale , G. Cao , Mater. Today 2016, 19, 109.

[13] Y. Gao , Z. Yan , J. L. Gray , X. He , D. Wang , T. Chen , Q. Huang , Y. C. Li , H. Wang , S. H. Kim , T. E. Mallouk , D. Wang , Nat. Mater. 2019, 18, 384. 30858569 10.1038/s41563-019-0305-8

[14] Q. Pang , X. Liang , I. R. Kochetkov , P. Hartmann , L. F. Nazar , Angew. Chem., Int. Ed. Engl. 2018, 57, 9795. 29947071 10.1002/anie.201805456

[15] R. Xu , X.‐Q. Zhang , X.‐B. Cheng , H.‐J. Peng , C.‐Z. Zhao , C. Yan , J.‐Q. Huang , Adv. Funct. Mater. 2018, 28, 1705838.

[16] X. Wang , Y. He , S. Liu , Y. Li , S. Tu , R. Zhan , Z. Chen , J. Fu , Z. Cai , L. Wang , Y. Sun , Adv. Funct. Mater. 2023, 35, 2307281.

[17] L. Suo , Y.‐S. Hu , H. Li , M. Armand , L. Chen , Nat. Commun. 2013, 4, 1481. 23403582 10.1038/ncomms2513

[18] J. Zheng , M. H. Engelhard , D. Mei , S. Jiao , B. J. Polzin , J.‐G. Zhang , W. Xu , Nat. Energy 2017, 2, 17012.

[19] X. Fan , L. Chen , O. Borodin , X. Ji , J. Chen , S. Hou , T. Deng , J. Zheng , C. Yang , S.‐C. Liou , K. Amine , K. Xu , C. Wang , Nat. Nanotechnol. 2018, 13, 715. 30013215 10.1038/s41565-018-0183-2

[20] J. Wang , Y. Yamada , K. Sodeyama , C. H. Chiang , Y. Tateyama , A. Yamada , Nat. Commun. 2016, 7, 12032. 27354162 10.1038/ncomms12032 PMC4931331

[21] Z. Zeng , V. Murugesan , K. S. Han , X. Jiang , Y. Cao , L. Xiao , X. Ai , H. Yang , J.‐G. Zhang , M. L. Sushko , J. Liu , Nat. Energy 2018, 3, 674.

[22] J. Alvarado , M. A. Schroeder , T. P. Pollard , X. Wang , J. Z. Lee , M. Zhang , T. Wynn , M. Ding , O. Borodin , Y. S. Meng , K. Xu , Energy Environ. Sci. 2019, 12, 780.

[23] Y. He , M. Zhang , A. Wang , B. Zhang , H. Pham , Q. Hu , L. Sheng , H. Xu , L. Wang , J. Park , X. He , ACS Appl. Mater. Interfaces 2022, 14, 33952. 10.1021/acsami.2c05801 35830236

[24] X. Wang , Y. He , S. Tu , L. Fu , Z. Chen , S. Liu , Z. Cai , L. Wang , X. He , Y. Sun , Energy Storage Mater. 2022, 49, 135.

[25] K. Yan , Z. Lu , H.‐W. Lee , F. Xiong , P.‐C. Hsu , Y. Li , J. Zhao , S. Chu , Y. Cui , Nat. Energy 2016, 1, 16010.

[26] Y. Han , B. Liu , Z. Xiao , W. Zhang , X. Wang , G. Pan , Y. Xia , X. Xia , J. Tu , InfoMat 2021, 3, 155.

[27] H. Shi , J. Qin , K. Huang , P. Lu , C. Zhang , Y. Dong , M. Ye , Z. Liu , Z.‐S. Wu , Angew. Chem., Int. Ed. 2020, 59, 12147. 10.1002/anie.202004284 32237031

[28] E. Markevich , G. Salitra , F. Chesneau , M. Schmidt , D. Aurbach , ACS Energy Lett. 2017, 2, 1321.

[29] K. Xu , Chem. Rev. 2014, 114, 11503. 25351820 10.1021/cr500003w

[30] Q.‐K. Zhang , X.‐Q. Zhang , J. Wan , N. Yao , T.‐L. Song , J. Xie , L.‐P. Hou , M.‐Y. Zhou , X. Chen , B.‐Q. Li , R. Wen , H.‐J. Peng , Q. Zhang , J.‐Q. Huang , Nat. Energy 2023, 8, 725.

[31] Q.‐K. Zhang , S.‐Y. Sun , M.‐Y. Zhou , L.‐P. Hou , J.‐L. Liang , S.‐J. Yang , B.‐Q. Li , X.‐Q. Zhang , J.‐Q. Huang , Angew. Chem., Int. Ed. 2023, 61, e202306889. 10.1002/anie.202306889 37442815

[32] X.‐B. Cheng , S.‐J. Yang , Z. Liu , J.‐X. Guo , F.‐N. Jiang , F. Jiang , X. Xiong , W. Bo Tang , H. Yuan , J.‐Q. Huang , Y. Wu , Q. Zhang , Adv. Mater. 2023, 2307370. 10.1002/adma.202307370 37684038

[33] L. Benitez , J. M. Seminario , J. Electrochem. Soc. 2017, 164, E3159.

[34] H. Guo , Y. Tian , Y. Liu , Y. Bai , J. Wu , F. Kang , B. Li , ACS Appl. Mater. Interfaces 2023, 15, 1201. 36576328 10.1021/acsami.2c17628

[35] X.‐X. Ma , X. Shen , X. Chen , Z.‐H. Fu , N. Yao , R. Zhang , Q. Zhang , Small Struct. 2022, 3, 2200071.

[36] A. Ramasubramanian , V. Yurkiv , T. Foroozan , M. Ragone , R. Shahbazian‐Yassar , F. Mashayek , J. Phys. Chem. C 2019, 123, 10237.

[37] J. Chen , X. Fan , Q. Li , H. Yang , M. R. Khoshi , Y. Xu , S. Hwang , L. Chen , X. Ji , C. Yang , H. He , C. Wang , E. Garfunkel , D. Su , O. Borodin , C. Wang , Nat. Energy 2020, 5, 386.

[38] X. Fan , X. Ji , F. Han , J. Yue , J. Chen , L. Chen , T. Deng , J. Jiang , C. Wang , Sci. Adv. 2018, 4, eaau9245. 30588493 10.1126/sciadv.aau9245 PMC6303121

[39] R. Xu , F. Han , X. Ji , X. Fan , J. Tu , C. Wang , Nano Energy 2018, 53, 958.

[40] C. Cui , C. Yang , N. Eidson , J. Chen , F. Han , L. Chen , C. Luo , P.‐F. Wang , X. Fan , C. Wang , Adv. Mater. 2020, 32, 1906427. 10.1002/adma.201906427 32058645

[41] Y. Lin , T. Wang , L. Zhang , X. Peng , B. Huang , M. Wu , T. Zhao , Nano Energy 2022, 99, 107395.

[42] W. Huang , H. Wang , D. T. Boyle , Y. Li , Y. Cui , ACS Energy Lett. 2020, 5, 1128.

[43] N. Piao , S. Liu , B. Zhang , X. Ji , X. Fan , L. Wang , P.‐F. Wang , T. Jin , S.‐C. Liou , H. Yang , J. Jiang , K. Xu , M. A. Schroeder , X. He , C. Wang , ACS Energy Lett. 2021, 6, 1839.

[44] J. Tan , J. Matz , P. Dong , J. Shen , M. Ye , Adv. Energy Mater. 2021, 11, 2100046.

[45] X. Ji , S. Hou , P. Wang , X. He , N. Piao , J. Chen , X. Fan , C. Wang , Adv. Mater. 2020, 32, 2002741. 10.1002/adma.202002741 33035375

[46] Y. Lu , Z. Tu , L. A. Archer , Nat. Mater. 2014, 13, 961. 25108613 10.1038/nmat4041

[47] M. He , R. Guo , G. M. Hobold , H. Gao , B. M. Gallant , Proc. Natl. Acad. Sci. U. S. A. 2020, 117, 73. 31848237 10.1073/pnas.1911017116 PMC6955333

[48] H.‐H. Sun , A. Dolocan , J. A. Weeks , R. Rodriguez , A. Heller , C. B. Mullins , J. Mater. Chem. A 2019, 7, 17782.

[49] T. Li , X.‐Q. Zhang , P. Shi , Q. Zhang , Joule 2019, 3, 2647.

[50] J. Jones , M. Anouti , M. Caillon‐Caravanier , P. Willmann , D. Lemordant , Fluid Phase Equilib. 2009, 285, 62.

[51] X.‐Q. Zhang , X.‐B. Cheng , X. Chen , C. Yan , Q. Zhang , Adv. Funct. Mater. 2017, 27, 1605989.

[52] S. Jiao , X. Ren , R. Cao , M. H. Engelhard , Y. Liu , D. Hu , D. Mei , J. Zheng , W. Zhao , Q. Li , N. Liu , B. D. Adams , C. Ma , J. Liu , J.‐G. Zhang , W. Xu , Nat. Energy 2018, 3, 739.

[53] S. J. Park , J. Y. Hwang , C. S. Yoon , H. G. Jung , Y. K. Sun , ACS Appl. Mater. Interfaces 2018, 10, 17985. 29701458 10.1021/acsami.8b04592

[54] P. Shi , F. Liu , Y. Feng , J. Zhou , X. Rui , Y. Yu , Small 2020, 16, 2001989. 10.1002/smll.202001989 32521092

[55] H. Wang , J. Zhang , H. Zhang , W. Li , M. Chen , Q. Guo , K. C. Lau , L. Zeng , G. Feng , D. Zhai , F. Kang , Cell Rep. Phys. Sci. 2022, 3, 100919.

[56] X.‐Q. Zhang , X. Chen , X.‐B. Cheng , B.‐Q. Li , X. Shen , C. Yan , J.‐Q. Huang , Q. Zhang , Angew. Chem., Int. Ed. 2018, 57, 5301. 10.1002/anie.201801513 29465827

[57] L.‐N. Wu , J. Peng , F.‐M. Han , Y.‐K. Sun , T. Sheng , Y.‐Y. Li , Y. Zhou , L. Huang , J.‐T. Li , S.‐G. Sun , J. Mater. Chem. A 2020, 8, 4300.

[58] S. Jurng , Z. L. Brown , J. Kim , B. L. Lucht , Energy Environ. Sci. 2018, 11, 2600.

[59] S. Li , D. Zhao , P. Wang , X. Cui , F. Tang , Electrochim. Acta 2016, 222, 668.

[60] S. Xiong , X. Kai , X. Hong , Y. Diao , Ionics 2012, 18, 249.

[61] S. Liu , Q. Zhang , X. Wang , M. Xu , W. Li , B. L. Lucht , ACS Appl. Mater. Interfaces 2020, 12, 33719. 32608965 10.1021/acsami.0c08094

[62] T. Yang , H. Zeng , W. Wang , X. Zhao , W. Fan , C. Wang , X. Zuo , R. Zeng , J. Nan , J. Mater. Chem. A 2019, 7, 8292.

[63] L. D. Ellis , I. G. Hill , K. L. Gering , J. R. Dahn , J. Electrochem. Soc. 2017, 164, A2426.

[64] Q. Lei , T. Yang , X. Zhao , W. Fan , W. Wang , L. Yu , S. Guo , X. Zuo , R. Zeng , J. Nan , J. Electroanal. Chem. 2019, 846, 113141.

[65] Y. Chen , W. Zhao , Q. Zhang , G. Yang , J. Zheng , W. Tang , Q. Xu , C. Lai , J. Yang , C. Peng , Adv. Funct. Mater. 2020, 30, 2000396.

[66] Y. Han , Y. Jie , F. Huang , Y. Chen , Z. Lei , G. Zhang , X. Ren , L. Qin , R. Cao , S. Jiao , Adv. Funct. Mater. 2019, 29, 1904629.

[67] Y. Zhou , M. Su , X. Yu , Y. Zhang , J.‐G. Wang , X. Ren , R. Cao , W. Xu , D. R. Baer , Y. Du , O. Borodin , Y. Wang , X.‐L. Wang , K. Xu , Z. Xu , C. Wang , Z. Zhu , Nat. Nanotechnol. 2020, 15, 224. 31988500 10.1038/s41565-019-0618-4

[68] M. A. Hope , B. L. D. Rinkel , A. B. Gunnarsdóttir , K. Märker , S. Menkin , S. Paul , I. V. Sergeyev , C. P. Grey , Nat. Commun. 2020, 11, 2224. 32376916 10.1038/s41467-020-16114-x PMC7203113

[69] Y. Xu , K. Dong , Y. Jie , P. Adelhelm , Y. Chen , L. Xu , P. Yu , J. Kim , Z. Kochovski , Z. Yu , W. Li , J. LeBeau , Y. Shao‐Horn , R. Cao , S. Jiao , T. Cheng , I. Manke , Y. Lu , Adv. Energy Mater. 2022, 12, 2200398.

[70] M. J. Zachman , Z. Tu , S. Choudhury , L. A. Archer , L. F. Kourkoutis , Nature 2018, 560, 345. 30111789 10.1038/s41586-018-0397-3

[71] Z. Yu , H. Wang , X. Kong , W. Huang , Y. Tsao , D. G. Mackanic , K. Wang , X. Wang , W. Huang , S. Choudhury , Y. Zheng , C. V. Amanchukwu , S. T. Hung , Y. Ma , E. G. Lomeli , J. Qin , Y. Cui , Z. Bao , Nat. Energy 2020, 5, 526.

[72] S. T. Oyakhire , W. Zhang , Z. Yu , S. E. Holmes , P. Sayavong , S. C. Kim , D. T. Boyle , M. S. Kim , Z. Zhang , Y. Cui , S. F. Bent , ACS Energy Lett. 2023, 8, 869.

[73] J. Wang , W. Huang , A. Pei , Y. Li , F. Shi , X. Yu , Y. Cui , Nat. Energy 2019, 4, 664.

[74] Z. Zhang , Y. Li , R. Xu , W. Zhou , Y. Li , S. T. Oyakhire , Y. Wu , J. Xu , H. Wang , Z. Yu , D. T. Boyle , W. Huang , Y. Ye , H. Chen , J. Wan , Z. Bao , W. Chiu , Y. Cui , Science (New York, N.Y.) 2022, 375, 66. 34990230 10.1126/science.abi8703

[75] W. Huang , P. M. Attia , H. Wang , S. E. Renfrew , N. Jin , S. Das , Z. Zhang , D. T. Boyle , Y. Li , M. Z. Bazant , B. D. McCloskey , W. C. Chueh , Y. Cui , Nano Lett. 2019, 19, 5140. 31322896 10.1021/acs.nanolett.9b01515

[76] Y. Li , Y. Li , A. Pei , K. Yan , Y. Sun , C.‐L. Wu , L.‐M. Joubert , R. Chin , A. L. Koh , Y. Yu , J. Perrino , B. Butz , S. Chu , Y. Cui , Science (New York, N.Y.) 2017, 358, 506. 29074771 10.1126/science.aam6014

[77] T. Liu , L. Lin , X. Bi , L. Tian , K. Yang , J. Liu , M. Li , Z. Chen , J. Lu , K. Amine , K. Xu , F. Pan , Nat. Nanotechnol. 2019, 14, 50. 30420761 10.1038/s41565-018-0284-y

[78] P. Sayavong , W. Zhang , S. T. Oyakhire , D. T. Boyle , Y. Chen , S. C. Kim , R. A. Vilá , S. E. Holmes , M. S. Kim , S. F. Bent , Z. Bao , Y. Cui , J. Am. Chem. Soc. 2023, 145, 12342. 37220230 10.1021/jacs.3c03195

[79] S. T. Oyakhire , H. Gong , Y. Cui , Z. Bao , S. F. Bent , ACS Energy Lett. 2022, 7, 2540.

[80] S. Yuan , S. Weng , F. Wang , X. Dong , Y. Wang , Z. Wang , C. Shen , J. L. Bao , X. Wang , Y. Xia , Nano Energy 2021, 83, 105847.

[81] Y.‐X. Yao , J. Wan , N.‐Y. Liang , C. Yan , R. Wen , Q. Zhang , J. Am. Chem. Soc. 2023, 145, 8001. 36988463 10.1021/jacs.2c13878

[82] X. Wang , M. Zhang , J. Alvarado , S. Wang , M. Sina , B. Lu , J. Bouwer , W. Xu , J. Xiao , J. G. Zhang , J. Liu , Y. S. Meng , Nano Lett. 2017, 17, 7606. 29090936 10.1021/acs.nanolett.7b03606

[83] Y. Li , Y. Li , A. Pei , K. Yan , Y. Sun , C. L. Wu , L. M. Joubert , R. Chin , A. L. Koh , Y. Yu , J. Perrino , B. Butz , S. Chu , Y. Cui , Science (New York, N.Y.) 2017, 358, 506. 29074771 10.1126/science.aam6014

[84] B. Han , X. Li , S. Bai , Y. Zou , B. Lu , M. Zhang , X. Ma , Z. Chang , Y. S. Meng , M. Gu , Matter 2021, 4, 3741.

[85] Z. L. Brown , S. Jurng , C. C. Nguyen , B. L. Lucht , ACS Appl. Energy Mater. 2018, 1, 3057.

[86] Z. Shadike , H. Lee , O. Borodin , X. Cao , X. Fan , X. Wang , R. Lin , S.‐M. Bak , S. Ghose , K. Xu , C. Wang , J. Liu , J. Xiao , X.‐Q. Yang , E. Hu , Nat. Nanotechnol. 2021, 16, 549. 33510453 10.1038/s41565-020-00845-5

[87] Y. Ji , J. Qiu , W. Zhao , T. Liu , Z. Dong , K. Yang , G. Zheng , G. Qian , M. Yang , Q. Chen , K. Amine , F. Pan , L. Yang , Chem 2023, 9, 2943.

[88] D. Aurbach , E. Pollak , R. Elazari , G. Salitra , C. S. Kelley , J. Affinito , J. Electrochem. Soc. 2009, 156, A694.

[89] S. Xiong , K. Xie , Y. Diao , X. Hong , Electrochim. Acta 2012, 83, 78.

[90] W. Li , H. Yao , K. Yan , G. Zheng , Z. Liang , Y.‐M. Chiang , Y. Cui , Nat. Commun. 2015, 6, 7436. 26081242 10.1038/ncomms8436

[91] E. Markevich , G. Salitra , D. Aurbach , ACS Energy Lett. 2017, 2, 1337.

[92] R. McMillan , H. Slegr , Z. X. Shu , W. Wang , J. Power Sources 1999, 81‐82, 20.

[93] E. Markevich , G. Salitra , F. Chesneau , M. Schmidt , D. Aurbach , ACS Energy Lett. 2017, 2, 1321.

[94] L. Chen , K. Wang , X. Xie , J. Xie , J. Power Sources 2007, 174, 538.

[95] D. Aurbach , K. Gamolsky , B. Markovsky , Y. Gofer , M. Schmidt , U. Heider , Electrochim. Acta 2002, 47, 1423.

[96] A. L. Michan , B. S. Parimalam , M. Leskes , R. N. Kerber , T. Yoon , C. P. Grey , B. L. Lucht , Chem. Mater. 2016, 28, 8149.

[97] K. Ushirogata , K. Sodeyama , Y. Okuno , Y. Tateyama , J. Am. Chem. Soc. 2013, 135, 11967. 23901789 10.1021/ja405079s

[98] A. L. Michan , B. S. Parimalam , M. Leskes , R. N. Kerber , T. Yoon , C. P. Grey , B. L. Lucht , Chem. Mater. 2016, 28, 8149.

[99] K. Kim , I. Park , S.‐Y. Ha , Y. Kim , M.‐H. Woo , M.‐H. Jeong , W. C. Shin , M. Ue , S. Y. Hong , N.‐S. Choi , Electrochim. Acta 2017, 225, 358.

[100] H. Shin , J. Park , A. M. Sastry , W. Lu , J. Electrochem. Soc. 2015, 162, A1683.

[101] T. Hu , J. Tian , F. Dai , X. Wang , R. Wen , S. Xu , J. Am. Chem. Soc. 2023, 145, 1327. 36576963 10.1021/jacs.2c11521

