# Supplementary material for: Insight into uniform filming of LiF‐rich interphase via synergistic adsorption for high‐performance lithium metal anode
Source: Exploration (Beijing). 2023 Dec 22;4(2):20230114. doi: 10.1002/EXP.20230114 (PMC11022620; doi:10.1002/EXP.20230114)
Supplement: Supplementary file 1 — Supporting Information [file EXP2-4-20230114-s001.docx]

Insight into Uniform Filming of LiF-rich Interphase via Synergistic Adsorption for High-performance Lithium Metal Anode

Yufang He^1^, Li Wang ^1, *^, Aiping Wang^1^, Bo Zhang^1^, Hiep Pham^2^, Jonghyun Park^2,*^, and Xiangming He^1, *^

^1^Institute of Nuclear and New Energy Technology, Tsinghua University, Beijing 100084, China

^2^Department of Mechanical Engineering and Aerospace Engineering, Missouri University of Science and Technology, Rolla, 65401, USA

*Email: wang-l@tsinghua.edu.cn; parkjonghy@mst.edu; [hexm@tsinghua.edu.cn](mailto:hexm@tsinghua.edu.cn)

**Keywords:** LiF-rich SEI; electrolyte-derived species; synergistic adsorption; film growth mechanism; lithium metal anode

**Method**

The theoretical calculation is conducted using Kohn-Sham DFT with the projector augmented wave method, which is applied in the Vienna Ab-initio Simulation Package (VASP)^1^. The generalized gradient approximation (GGA) of Perdew-Burke-Ernzerhof (PBE) is used to approximate the exchange-correlation functional and the projector augmented wave (PAW) method is used for electron and core interaction^2-4^. The electron states are sampled using a k-point grid of 3 × 3× 1 centered at the origin. The electronic wave functions are expanded in a plane wave basis set with an energy cutoff of 400 eV. The criteria for ionic relaxation and electronic self-consistent are set to be 10^-4^ eV and 0.03 eV/Å respectively. The Van der Waals interactions are considered by using the DFT-D3 method with Becke-Jonson damping^5^ and the dipole correction is applied to the slab structures as well.

To calculate the adsorption energy of individual LiF, LiNO_2_, Li_2_O, Li_3_N, LiPO_2_F_2_, and Poly(VC) absorbed on lithium metal anode (LMA) surface, the structure of individual LiF, LiNO_2_, Li_2_O, Li_3_N, LiPO_2_F_2_, and Poly(VC) adsorption on the LMA surface is constructed. The absorption energy is the energy needed for the species to be absorbed on the substrate. For example, the adsorption energy of LiF can be expressed as follows^6^:

$E_{\mathrm{adsorb}}=E_{Li metal+LiF}-(E_{Li metal}+E_{\mathrm{LiF}})$ (1)

(Where E_Li metal+LiF_ is the total energy of the slab structure with one LiF absorbed on the Li metal surface, E_Li metal_ is the total energy of the Li metal surface structure, and E_LiF_ is the total energy of one LiF.)

To investigate the synergetic precipitation of electrolyte-derived inorganic LiN_x_O_y_, Li_x_PO_y_F_z_, and organic poly (VC) on the LiF adsorption, deposition, and film growth mode, the structures of LiF/LiF/Li, LiF/LiNO_2_/ Li, LiF/LiPO_2_F_2_/ Li, and LiF/Poly (VC)/Li are constructed. For instance, the adsorption energy of LiNO_2_ and LiF can be described as follows:

$E_{\mathrm{adsorb}}=E_{Li metal+LiF+LiNO2}-(E_{\mathrm{LiF}}+E_{Li metal+LiNO2})$ (2)

(Where E_Li_ _metal+LiF+LiNO2_ is the total energy of the slab structure with LiF and LiNO_2_ absorbed on the Li metal surface, E_LiF_ is the total energy of one LiF, and $E_{Li metal+LiNO2}$ is the total energy of the slab structure with one LiNO_2_ adsorbed on the Li metal surface.)


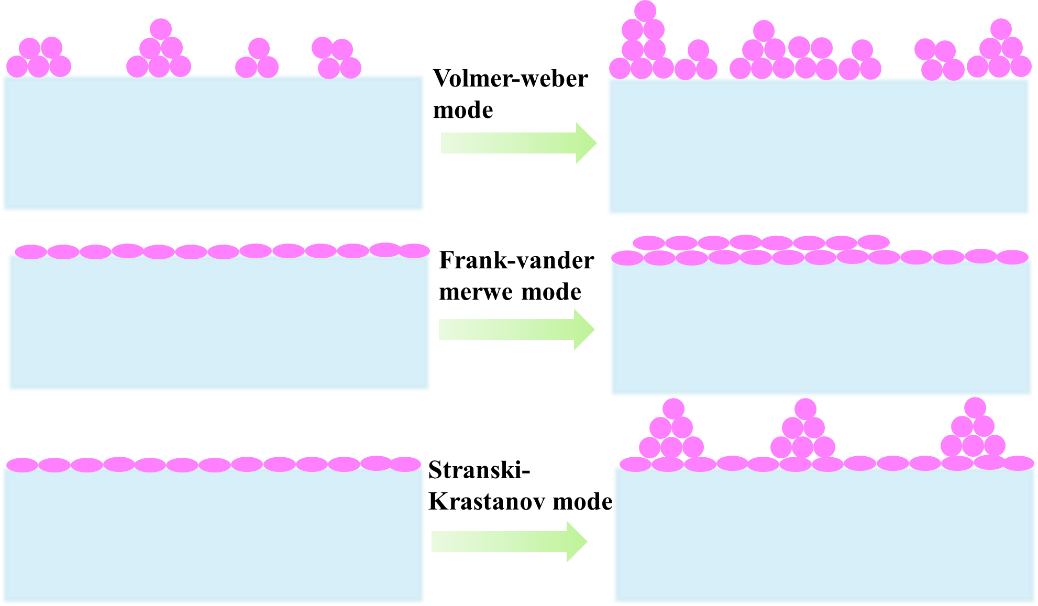


Figure S1. The schematic of three main film growth modes: Volmer-Weber mode (VWM), Frank-Vander Merwe mode (FVDM), and Stranski-Krastanov mode (SKM).


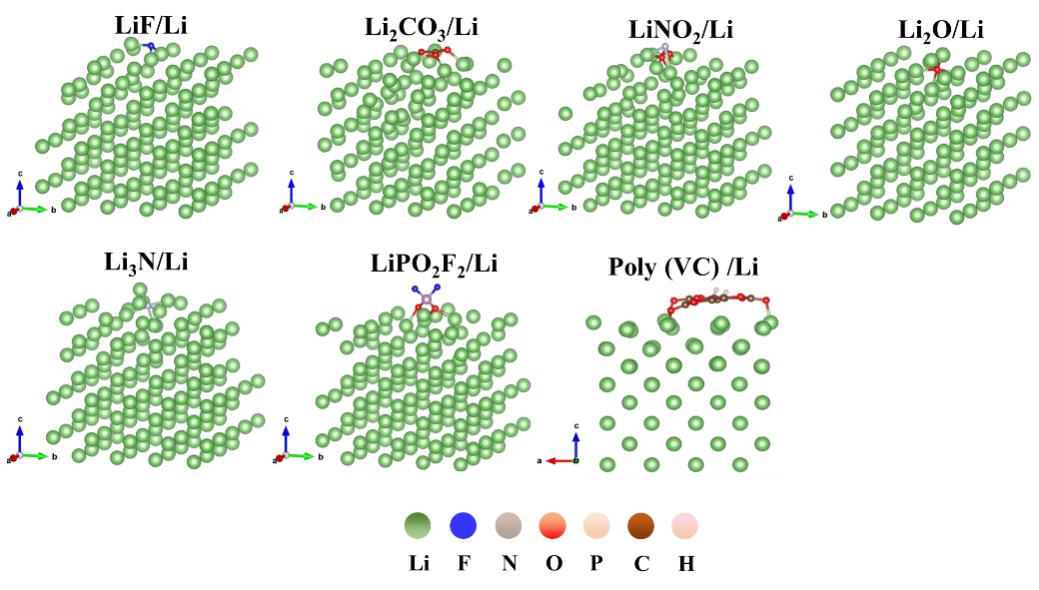


Figure S2. The optimized structures for LiF/Li, Li_2_CO_3_/Li, Li_2_O/Li, LiNO_2_/Li, Li_3_N/Li, LiPO_2_F_2_/Li, and Poly(VC)/Li.


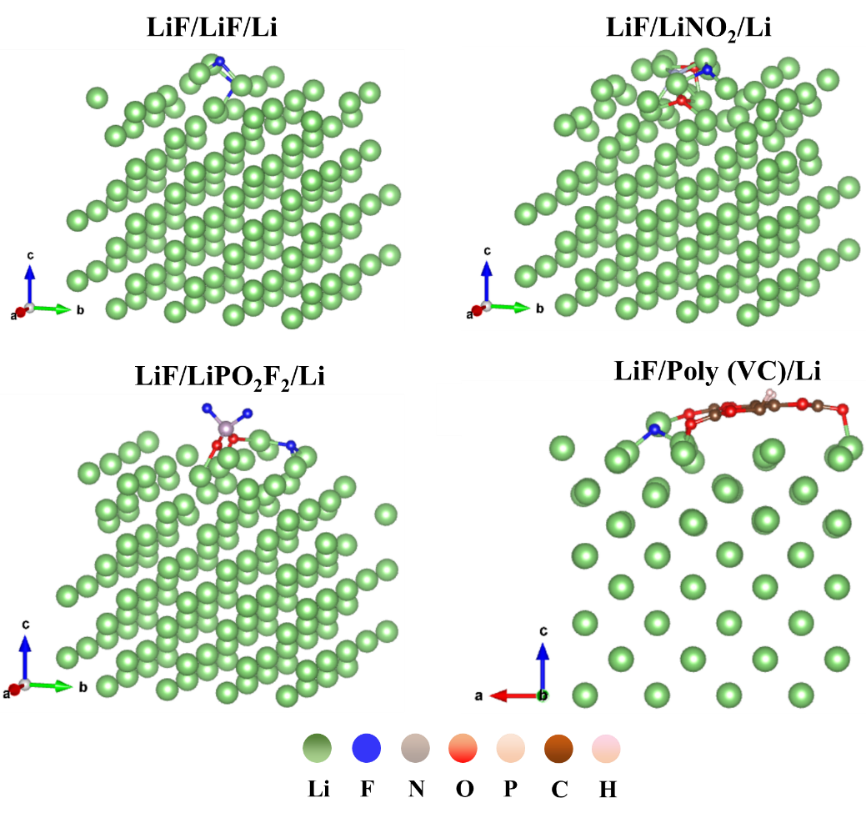


(a)


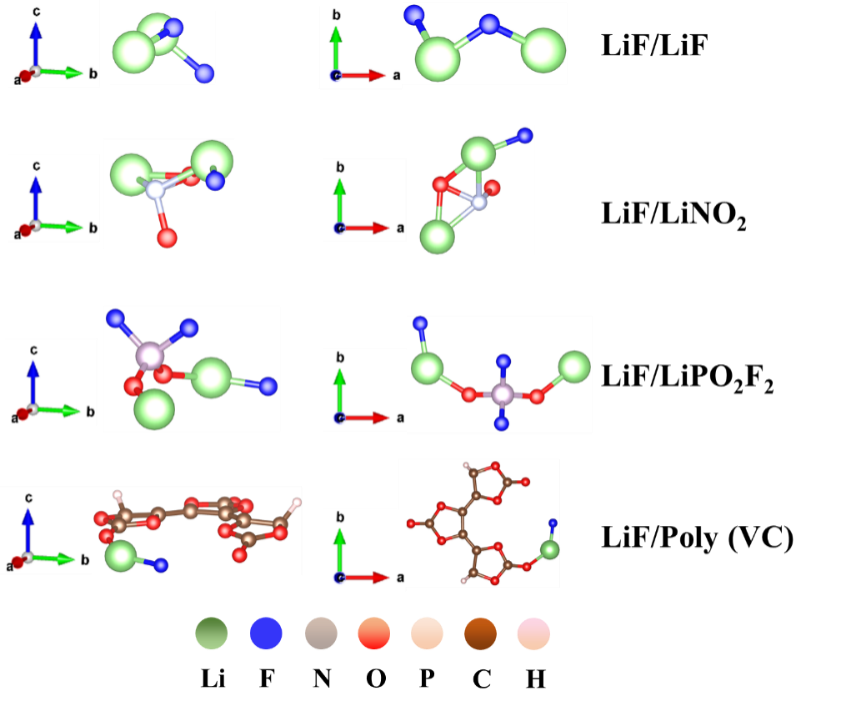


(b)

Figure S3. The optimized structures of LiF/LiF/Li, LiF/LiNO_2_/Li, LiF/LiPO_2_F_2_/Li, and LiF/Poly(VC)/Li (a); The enlarged atomic structure of LiF/LiF/Li, LiF/LiNO_2_/Li, LiF/LiPO_2_F_2_/Li, and LiF/Poly(VC)/Li in different views (b). It clearly shows that the inorganic LiNO_2_, LiPO_2_F_2_, and organic poly (VC) promote uniform LiF adsorption and deposition on the LMA surface via forming the Li-O bond.

**Reference**

[1] Kresse, G.; Furthmüller, J., Efficiency of ab-initio total energy calculations for metals and semiconductors using a plane-wave basis set. Computational Materials Science 1996, 6 (1), 15-50.

[2] Perdew, J. P.; Burke, K.; Ernzerhof, M., Generalized Gradient Approximation Made Simple. Physical review letters 1996, 77 (18), 3865-3868.

[3] Blöchl, P. E., Projector augmented-wave method. Physical review. B, Condensed matter 1994, 50 (24), 17953-17979.

[4] Kresse, G.; Joubert, D., From ultrasoft pseudopotentials to the projector augmented-wave method. Physical Review B 1999, 59 (3), 1758-1775.

[5] Grimme, S.; Ehrlich, S.; Goerigk, L., Effect of the damping function in dispersion corrected density functional theory. Journal of computational chemistry 2011, 32 (7), 1456-65.

[6] Wang, X.; He, Y.; Tu, S.; Fu, L.; Chen, Z.; Liu, S.; Cai, Z.; Wang, L.; He, X.; Sun, Y., Li plating on alloy with superior electro-mechanical stability for high energy density anode-free batteries. Energy Storage Materials 2022, 49, 135-143.
